# Insights into the mechanism of human papillomavirus E2-induced procaspase-8 activation and cell death

**DOI:** 10.1038/srep21408

**Published:** 2016-02-24

**Authors:** Nitu Singh, Sanjib Senapati, Kakoli Bose

**Affiliations:** 1 Integrated Biophysics and Structural Biology (IBSB) Laboratory, Advanced Centre for Treatment, Research and Education in Cancer (ACTREC), Navi Mumbai, India; 2 Department of Biotechnology, Office No. 503, Lab No. 510, Indian Institute of Technology Madras, Adyar, Chennai, 600036, India.

## Abstract

High-risk human papillomavirus (HR-HPV) E2 protein, the master regulator of viral life cycle, induces apoptosis of host cell that is independent of its virus-associated regulatory functions. E2 protein of HR-HPV18 has been found to be involved in novel FADD-independent activation of caspase-8, however, the molecular basis of this unique non-death-fold E2-mediated apoptosis is poorly understood. Here, with an interdisciplinary approach that involves *in silico*, mutational, biochemical and biophysical probes, we dissected and characterized the E2-procasapse-8 binding interface. Our data demonstrate direct non-homotypic interaction of HPV18 E2 transactivation domain (TAD) with α2/α5 helices of procaspase-8 death effector domain-B (DED-B). The observed interaction mimics the homotypic DED-DED complexes, wherein the conserved hydrophobic motif of procaspase-8 DED-B (F122/L123) occupies a groove between α2/α3 helices of E2 TAD. This interaction possibly drives DED oligomerization leading to caspase-8 activation and subsequent cell death. Furthermore, our data establish a model for E2-induced apoptosis in HR-HPV types and provide important clues for designing E2 analogs that might modulate procaspase-8 activation and hence apoptosis.

Viruses have evolved complex strategies to selectively manipulate host apoptotic machinery to their advantage. They specifically inhibit or induce apoptosis for efficient replication, evasion of host defence mechanisms and viral propagation. Oncogenic high-risk human papillomaviruses (HR-HPVs) display antiapoptotic activities via oncoprotein E6, which interfere with both intrinsic and extrinsic pathways by targeting p53^1^ and FADD^2^ for degradation. However, the proapoptotic role has been attributed to E2 protein that acts as a central regulator for viral replication and transactivation of essential oncogenes^3^,^4^. Strikingly, involvement of E2 in apoptosis is specific and conserved only in the HR-HPVs^5^,^6^, where this activity is speculated to be relevant for the vegetative viral cycle, which is closely linked to the differentiation of the infected keratinized epithelia. The E6 and E7 oncoproteins of HR-HPVs interfere with cell cycle exit and block cellular differentiation, thereby inducing greater resistance to apoptosis^7^. During the natural course of infection with HR-HPVs, it is speculated that the E2 apoptotic activity could counteract these effects of E6 and E7, contrary to the low risk infected cells where many of the E6 and E7 functions are non-conserved^8^,^9^. It is proposed that accumulation of E2 protein at a threshold level in the course of keratinocyte differentiation triggers classical apoptosis, facilitating virus release. The proapoptotic activity of E2 proteins, however, would also explain the disruption of E2 gene post viral genome integration into the host chromosome in HPV-associated cervical carcinoma^10^. Furthermore, several studies have found that E2-mediated apoptosis is independent of its virus-associated activities and is not cell-type specific^11^,^12^. The transactivation domain (TAD) of E2 induces activation of apical caspase-8 that is independent of its transcriptional functions^5^,^12^. Although its mechanism of apoptosis induction is not very clear, literature on HPV18 E2 suggest its involvement in direct interaction with procaspase-8, a member of death inducing signaling complex (DISC) of the extrinsic apoptotic pathway^13^.

DISC assembly is achieved through the homotypic interaction driven by the death-fold domains including death domain (DD) and death effector domain (DED)^14^,^15^,^16^. Ligation of Fas or TRAIL receptors recruits adaptor protein FADD (Fas-associated death domain) via homotypic interaction between DD within the receptor and FADD. The free N-terminal DED of FADD then recruits DED-only proteins including procaspase-8 or -10, forming an active DISC. This enables proximity-induced activation of initiator proenzymes leading to oligomerization, zymogen processing and downstream cascade activation^17^,^18^. The death or pseudo-death domains (DD/DED) that are signatures of these adaptor molecules are considered as prerequisites for caspase-8 activation^19^,^20^. However, HPV18 E2 TAD, despite lacking the six α-helical bundle structure of a typical death domain, alone is sufficient to induce caspase-8 activation through direct interaction with procaspase-8 prodomain (containing tandem DEDs; DED-A and DED-B)^13^. This FADD-independent interaction thus represents a novel non-adaptor-mediated caspase activation pathway, the molecular and structural determinants of which have remained elusive till date. Therefore, understanding the driving force for this interaction and molecular mechanism of the novel cell death pathway raises possibility of harnessing it for therapeutic interventions especially in conditions where caspase activation is prevented either due to genetic inactivation of the receptor or adaptor proteins.

In the present study, we demonstrate that E2 specifically interacts with DED-B domain of procaspase-8 thus driving caspase-8 self-oligomerization and its subsequent activation. This study that include *in-silico*, protein engineering, biophysics and cell biology tools, reveals the molecular basis of novel E2-procaspase-8 interaction and hence provides a model for E2-induced apoptosis in high-risk HPV types.

## Results

### Mapping the minimal HPV18 E2 – procaspase-8 interaction regions

**Figure 1:**
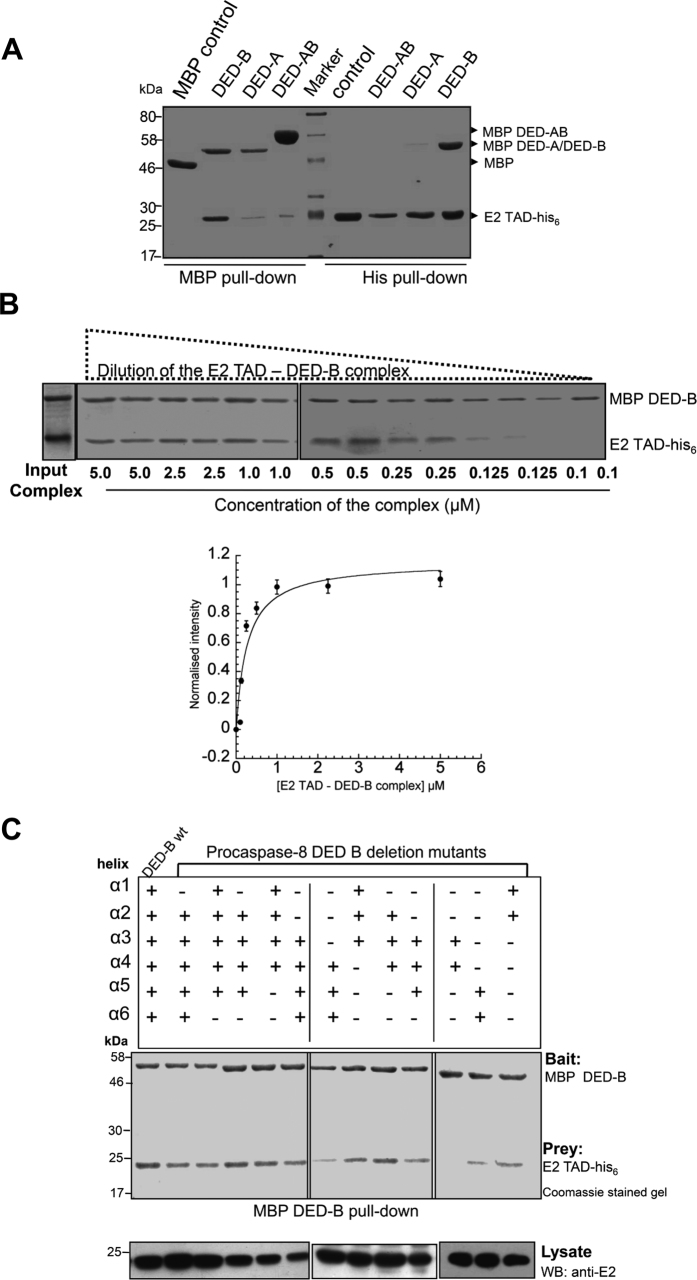
Mapping binding region of E2-procaspase-8 interaction. (**A**) Pull-down of E2 TAD and procasapse-8 DED domains. Lanes 1–4 represent MBP pull-down using amylose resin while lanes 6–9 is for reverse his_6_ pull-down with Ni^2+^-IDA resin. In lane-1, MBP was checked for binding to E2 TAD-his_6_, while in lanes 2–4, MBP fused DED-B, DED-A and DED-AB acted as baits. In lanes 6–9, E2 TAD-his_6_ acted as a bait to monitor the binding of MBP, MBP-tagged DED-AB, DED-A and DED-B respectively. The image is of 12% SDS-PAGE Coomassie-stained gel. (**B**) Dilution experiment with the isolated E2 TAD – DED-B complex. *Top panel*-The complex diluted to different final concentrations as indicated were analyzed on SDS-PAGE. The lower panel shows a plot derived from quantitative analysis of background corrected band intensity measured as E2 TAD to DED-B ratio for various dilutions of the complex. The measured intensity is represented as normalized intensity for the data obtained from three independent complex preparations and their dilutions. The error bars show the standard error. (**C**) MBP pull-down assay of deletion constructs with MBP DED-B and E2 TAD-his_6_ as bait and prey respectively. 20 μg of the total cell lysate was immunoblotted with anti-E2 antibody to confirm the presence of E2 protein in each sample.

It has been previously demonstrated that the HPV18 E2 TAD binds directly to the tandem DED of procaspase-8 to induce apoptosis^13^. Since isolated procaspase-8 DEDs demonstrate strong tendency to aggregate and exhibit lack of solubility in buffers with different salt concentration and detergents^21^,^22^, we developed a bacterial co-expression system for interaction analyses. The individual genes, procaspase-8 tandem DEDs, DED-A, DED-B and E2 TAD, were sub-cloned in two compatible plasmid sets with different origin of replication, antibiotic selection, inducer and affinity-tag. HPV18 E2 TAD was cloned in an ara-operon plasmid facilitating expression of C-terminal his_6_-tagged E2 TAD. Similarly, lac-operon constructs encoding maltose binding protein (MBP)-fused DEDs were cloned in pMALc5-E-TEV vector. The constructs generated are illustrated in Supplementary Fig. S1A. The proteins were co-expressed in *E. coli* BL21 (DE3) cells and pull-down assay with appropriate controls was performed as described in the experimental section. *In vitro* pull-down of the complex using amylose resin having affinity for MBP-tagged DED proteins demonstrated that E2 TAD-his_6_ binds faintly to DED-AB or DED-A, while it strongly associates with DED-B (Fig. 1A). Similar results were obtained when we performed a reverse E2 TAD-his_6_ pull-down of the complex using Ni^+2^ iminodiacetic acid (IDA) resin. These observations suggest that HPV18 E2 TAD mediates direct interaction with DED-B of procasapase-8 prodomain. It is interesting to note that despite having domain-B, DED-AB did not show any significant interaction with E2 TAD.

Since the biochemical analyses of DED complex is limited due to lack of solubility, a dilution experiment was performed with the isolated TAD – DED-B complex as previously demonstrated for Fas–FADD death domain complex^23^. It was observed that the complex exhibited a cooperative dissociation below 500 nM concentration, based upon plot derived from quantitative SDS–PAGE analysis from various TAD – DED-B complex dilutions (see Fig. 1B) suggesting very high affinity between the two proteins. Furthermore, the complex was also found to be stable in buffers of different ionic strengths (Supplementary Fig. S2). Next, to map the minimal binding region of DED-B, 12 deletion constructs comprising different combination of six α-helices were generated as shown in Supplementary Fig. S1B. Pull-down of the complexes using MBP-fused DED-B proteins as bait and E2 TAD-his_6_ as prey was performed using amylose resin, and it was observed that the combined α2 and α5 helices deletion construct showed disruption of interaction with E2 TAD (Fig. 1C). These results highlight importance of these helices in mediating E2 TAD – DED-B interaction.

### *In silico* prediction of E2 – procaspase-8 binding interface

Using the homology-modeled structure for procaspase-8 DED and E2 TAD, docking analysis was undertaken to identify the binding interface. Recently, the crystal structure of procaspase-8 DED-AB (F122A, I128D) has been reported^24^. Superposition of our modeled and the available DED-AB crystal structure (PDB ID. 4ZBW) demonstrates a root mean square deviation of less than 1.2 Å, thus attesting the authenticity of our model. The inter-molecular interface of the docked complex had 1667 Å^2^ of extensively buried surface area that notably involved residues F122, L123, D158, K161, R162, Q166 from DED-B and Q35, Q38, R41, W42, F48, D52, I77, Q80, M81, Q84 from E2 TAD (see Supplementary Fig. S3). Strikingly, 80% of the interactions involved residues from α2 and α5 of DED-B, which very well corroborates with the deletion studies. For E2 TAD, 60% of the interactions involved residues from α2 helix thus emphasizing its importance in E2-mediated apoptosis. This observation is in accordance with a previous finding where deletion of helix α2 from E2 showed diminished apoptosis^13^.

### Identification of procaspase-8 specific binding site on E2

**Figure 2:**
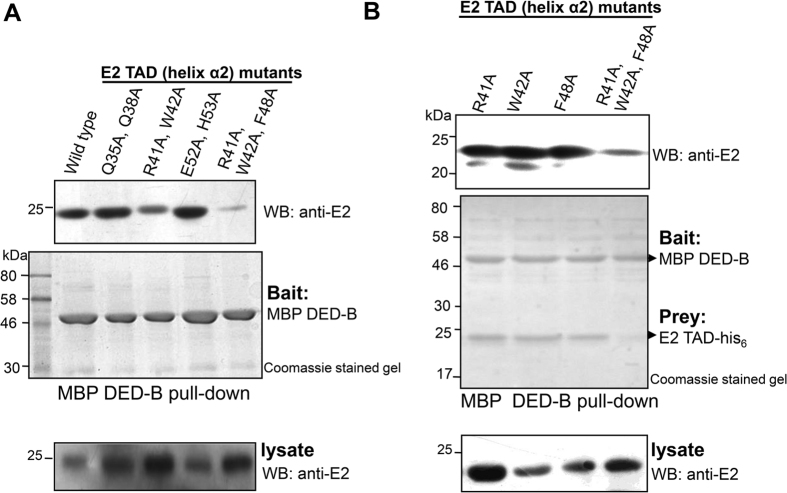
Identification of E2 binding surface for procaspase-8. (**A**,**B**) MBP pull-down assay for E2 wild-type and mutants with procaspase-8 DED-B. E2 TAD wild-type or mutants (prey) as indicated were tested for their binding to MBP-tagged DED-B (bait) using amylose affinity purification. 20 μg of the total lysate immunoblotted with anti-E2 confirmed the presence of E2 TAD in all the samples. The varying amount of protein in the total bacterial lysate is most likely due to difference in the expression level of these proteins in the cell. Although lower in case of single mutants, the *in vitro* isolated complexes for both the wild-type and single mutant proteins were similar, whereas the combined mutations of these residues significantly affected the complex formation.

To identify the key residues involved in the interaction, alanine scanning mutagenesis, a method of systematic alanine substitution, was performed. A series of single and combinatorial mutations in the α-2 helix of E2 TAD were generated followed by co-expression, and pull-down of TAD - DED-B complex using amylose resin. As shown in Fig. 2A, substituting set of residues comprising R41, W42 and F48 significantly affected its interaction with procaspase-8 DED-B. However, none of these point mutations alone inhibited the complex formation (Fig. 2B), suggesting an extensive array of binding network between E2 and procaspase-8. Generally, mutations interfere with the normal functions of any protein either by destabilizing the structure or perturbing a functional epitope that is crucial for binding to other macromolecule/ligand. Notably, these potential residues in E2 TAD are solvent exposed (Supplementary Table S1), and are thus unlikely to affect the structure. To confirm this, secondary and tertiary structural analyses was performed using far-UV circular dichroism and fluorescence spectroscopy respectively (see Supplementary Fig. S4), which demonstrated that the overall structural conformation of the mutants was similar to the wild-type TAD. Based on these results we conclude that interaction of E2 with procaspase-8 involves helix α-2 residues R41, W42 and F48.

### *Ex vivo* analysis of E2- procaspase-8 interaction

**Figure 3:**
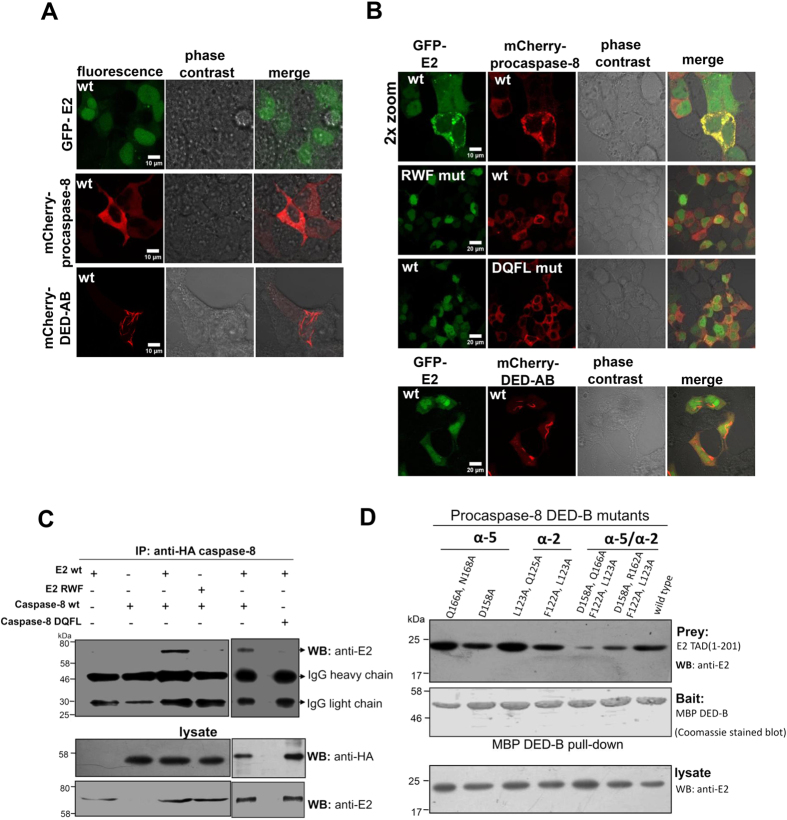
E2-procaspase-8 interaction and colocalization. (**A**,**B**) Expression and localization of catalytically inactive procaspase-8 full length, prodomain and HPV18 E2 in transfected cells. HEK293 cells were transfected with the indicated expression constructs and live-cell confocal images were acquired post 20–24 h of transfection. The abbreviations RWF and DQFL stand for: R41A/W42A/F48A and D158A/Q166A/ F122A/L123A respectively. Of the ~100 cells visualized, >45% cells expressing both the wild-type proteins showed re-distribution into co-localized cytoplasmic punctate structures, while it was significantly less, approximately 4–5% for cells expressing the mutant protein. Representative images for each of the transfected population are shown along with the scale bar. (**C**) Immunoprecipitation of E2-procaspase-8 complex. Extracts from transfected HEK293 cells were immunoprecipitated using an anti-HA antibody followed by immunoblotting using anti-E2. To check the presence of the proteins in the cell extract, 20 μg of the lysate was probed with anti-HA and anti-E2 antibodies. (**D**) MBP pull-down assay for wild-type E2 TAD and procaspase-8 DED-B mutants. Using amylose resin, MBP-tagged procaspase-8 DED mutants (bait) as indicated were tested for their binding to E2 TAD-his_6_ (prey) by probing with anti-E2. 20 μg of the total lysate was immunoblotted with anti-E2 to test the presence of E2 TAD in all the samples.

It has been previously established that ectopic expression of tagged/untagged HPV18 E2 or caspase-8 separately show nucleo-cytoplasmic distribution and diffused cytoplasmic localization respectively^5^. To study the relationship between E2 and caspase-8, and the importance of the interface residues, we ectopically co-expressed GFP-E2 and catalytically inactive mCherry-procaspase-8 C360A mutant so as to prevent induction of cell death upon interaction. Post 24 h of transfection in HEK293, live-cell images were captured by laser confocal microscope to visualize their localization. As anticipated, expression of either GFP-E2 or mCherry-procasapase-8 showed nucleo-cytoplasmic and diffused cytoplasmic localization respectively (Fig. 3A). However, co-expression of wild-type procaspase-8 and E2 resulted in redistribution of the two proteins into co-localized cytoplasmic punctate structures (Fig. 3B). Interestingly, this failed to happen in presence of E2 triple mutant (R41A/W42A/F48A) denoted as RWF, where caspase-8 showed diffused distribution while E2 exhibited its characteristic nucleo-cytoplasmic localization. To further substantiate these findings, HA-tagged procaspase-8 and GFP-E2 wild-type or triple mutant were co-transfected into HEK293 and the cell lysate was immunoprecipitated using anti-HA antibody. Immunoblot analysis using anti-E2 showed that E2 wild-type associates with procaspase-8, while E2 triple mutant does not show any interaction (Fig. 3C).

### Identification of E2-specific binding site on procaspase-8

A closer look into the available DED family complex reveals a large interaction surface^25^, which often lacks the presence of defined interaction sites as usually observed in regulatory complexes^26^,^27^. Our docking and deletion studies show a wide surface area of the interface involving helices α2/α5 of DED-B that is critical in mediating E2-procaspase-8 interaction. Therefore, to identify the key residues, series of alanine substitutions were carried out individually and in combination on these two helices. Pull-down of the complex using MBP-tagged procaspase-8 as bait and wild-type E2 TAD as prey showed that substitution of residues F122, L123 (helix α2b) and D158, Q166 (helix α5b) in combination significantly affected the interaction (Fig. 3D). Further, in co-localization studies, we observed that mCherry-procaspase-8 quadruple mutant (F122A/L123A/D158A/Q166A), indicated as DQFL, did not redistribute into the typical cytoplasmic punctate structure in presence of E2, as seen in case of wild-type complex (Fig. 3B). In addition, no co-immunoprecipitation was observed for the procaspase-8 quadruple mutant (Fig. 3C), thus confirming the importance of these residues in E2 – procaspase-8 interaction.

It is well established in the literature that procaspase-8 DED-AB over expression leads to formation of cytoplasmic filamentous structure called ‘death effector filaments’ (DEF)^28^, as shown in Fig. 3A. Recently, we reported that two conserved hydrophobic patches ‘LXXϕ’ and ‘FL’-motifs on the opposite surfaces of DED-A and DED-B, respectively are essential for DED-AB self-association and DEF formation^22^. Importantly, the FL-motif on DED-B corresponds to residues F122/L123, the patch that we identified to be involved in E2-procaspase-8 interaction (see Fig. 3). Therefore, we speculated that DED-AB self-association and E2 binding site on procaspase-DED do overlap, thus making the binding site unavailable. This might explain the seemingly paradoxical observation that DED-AB does not interact with E2 TAD, as shown in Fig. 1A. To test this, we co-expressed mCherry-fused DED-AB and GFP-E2 in HEK293 cells. Live-cell imaging of the co-transfected cells demonstrated that DED-AB forms cytoplasmic filaments without any colocalization with E2 (Fig. 3B). Furthermore, these results also indirectly validate the importance of F122/L123 residues of procaspase-8 for interaction with HPV18 E2.

### Interaction between E2 and adaptor protein FADD

**Figure 4:**
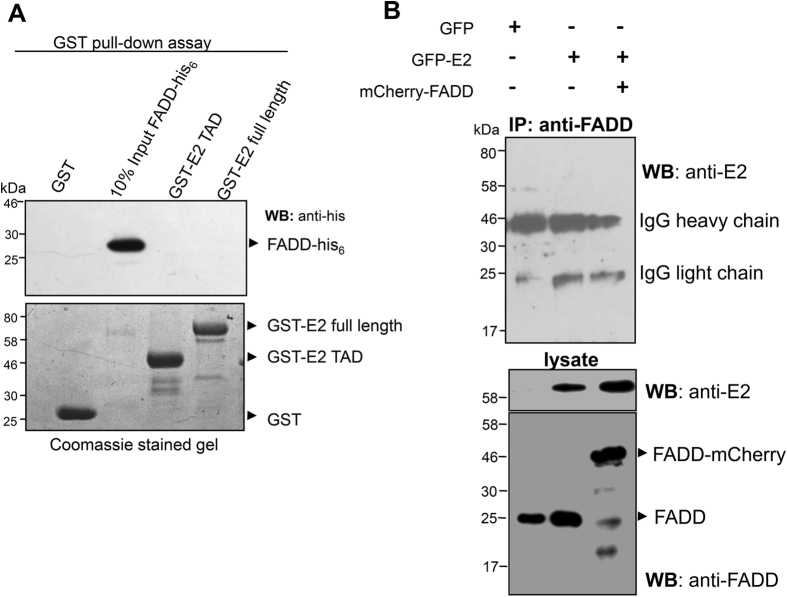
E2-FADD interaction analysis. (**A**) Upper panel: Western blot analysis of GST pull-down assay of HPV18 E2 full length or TAD proteins (bait) with recombinant FADD-his_6_ (prey). Lower panel shows the Coomassie-stained gel for the recombinant GST and GST-fusion proteins. (**B**) Immunoprecipitation of FADD. Extracts from HEK293 cells co-transfected as described were immunoprecipitated using an anti-FADD antibody followed by immunoblotting using anti-E2. 20 μg of the cell lysate was immunoblotted with anti-FADD and anti-E2 antibodies, to confirm the presence of these proteins in the cellular lysate.

Previous studies have reported that interaction between HPV18 E2 and procaspase-8 is independent of adaptor protein FADD^12^,^13^. However, to test whether there is any direct interaction between E2 and FADD, *in vitro* GST pull-down assay was performed with recombinant FADD-his_6_ and GST-fused wild-type E2 full length or TAD (Fig. 4A). We observed that FADD did not show direct interaction with the E2 protein. These observations were further validated by co-immunoprecipitation of ectopically expressed GFP-E2 and mCherry-fused FADD or endogenous FADD (Fig. 4B). As anticipated, no interaction was observed, thus confirming that E2 is not involved in the interaction with adaptor protein FADD.

### Monitoring the effect of mutations in E2 interface on cell death

**Figure 5:**
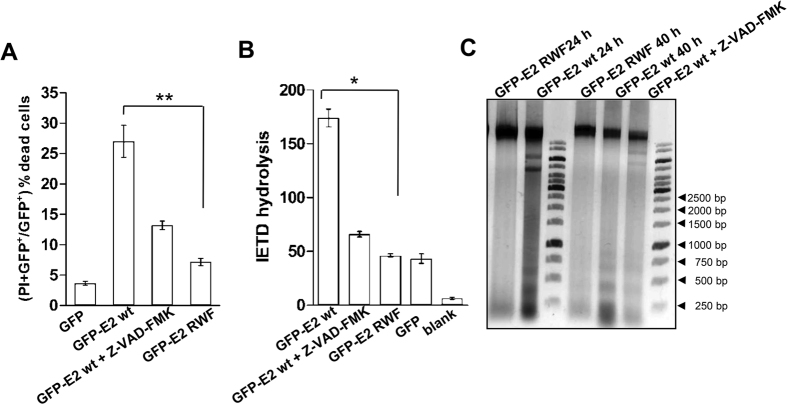
E2-induced cell death analyses. (**A**) Percentage of GFP-positive HEK293 dead cells expressing the indicated plasmids was quantified by propidium iodide uptake as described in the Methods section. (**B**) Caspase-8 activity was measured for the crude extracts isolated from the cells transfected with the plasmids as indicated, using fluorogenic Ac-IETD-AFC substrate. The bars and the error bars represent mean and standard deviation respectively. Statistical analysis using Student’s t-test, *p < 0.05 (**A**) and p < 0.01 (**B**). (**C**) Agarose gel electrophoresis of apoptotic DNA fragments. Total DNA extracted from transfected HEK293 cells was loaded on a 2% agarose gel. A typical DNA ladder consisting of multiples of 180 bp fragments was observed in case of wild-type E2 expressing cells while it was significantly reduced in mutant cells (M stands for 1 kilo-bp DNA marker). In each experiment, cells expressing wild-type E2 incubated with pan-caspase inhibitor (Z-VAD-FMK) was kept as experimental control to monitor that inhibition of the caspases affected substrate cleavage and apoptosis.

E2 proteins of high-risk HPVs induce apoptosis in all human cell types, including transformed and primary epithelial cells^3^,^11^,^12^. To test the effect of loss of E2-procaspase-8 interaction, cell death assays were performed in the presence of wild-type or triple mutant (RWF) E2 proteins in HEK293 cells. As shown previously^5^,^12^,^13^, for each experiment, cells expressing wild-type E2 incubated with pan-caspase inhibitor (Z-VAD-FMK) was used as the control to show that inhibition of the caspases affected substrate cleavage and cell viability. When ectopically expressed, wild-type GFP-E2 induced cell death as measured by propidium iodide uptake, while overexpression of GFP alone did not. In addition, the inhibitor reduced the level of E2-induced cell death, as anticipated. Strikingly, expression of GFP-E2 RWF mutant showed a significant reduction in percent cell death (Fig. 5A). These results highlight the importance of E2-procapase-8 interaction for caspase-8 activation and hence cell death. To measure caspase-8 activation, we determined the rate of hydrolysis of caspase-8 specific fluorogenic substrate IETD-AFC. It was found that the IETDase activity was readily detectable in extracts from wild-type GFP-E2-expressing cells but not in the mutant cells (Fig. 5B). Similar results were obtained when wild-type and mutant E2 were assayed by transfection into HeLa cells (see Supplementary Fig. S5). One of the characteristic events of apoptosis involves activation of endogenous endonucleases, resulting in DNA fragmentation. As shown in Fig. 5C, a typical ladder-like pattern, consisting of bands in the multiples of 180 base pairs (bp), could be observed for DNA extracted from wild-type E2-expressing cells post 24 and 40 h. However, substantially reduced fragments were observed from cells expressing mutant E2 protein or inhibitor controlled wild-type expressing cells. Overall, these results confirm that loss of E2-procaspase-8 interaction significantly decreases the apoptotic activity of E2 protein.

## Discussion

Complexities in molecular mechanisms of cancer have led toward search for new molecules for multi-targeted intervention. Therefore, understanding ways to modulate apoptotic pathway in cancer cells provides immense possibility for devising therapeutic strategies against the deadly disease. Despite several reports on novel apoptotic-inducer E2 protein, little is known about the structural basis and protein-protein interactions involved in mediating this function. Here, we have determined the molecular basis of E2-procaspase-8 interaction and its role in adaptor-independent caspase-8 activation and cell death.

Interaction analysis using series of mutants demonstrated that the residues from α2 helix of HPV18 E2 TAD mediate direct non-homotypic interaction with α2/α5 helices of procaspase-8 DED-B. The interface largely contains several non-bonded contacts and involves residues that spread out over an extended range of primary amino acid sequence. Furthermore, dilution experiment highlighted that the complex is highly stable even at lower protein concentrations, and in different buffer conditions. These observations possibly explain as to why we found it difficult to achieve complete abrogation of this interaction by simple deletion or point mutagenesis. Homotypic interactions among the death-fold domain proteins typically involve characteristic helix/helix - helix/helix association, where FL-motif from one DED is accommodated in the hydrophobic groove between α1 and α4 helices of another^22^,^29^. Our data suggests that interaction of E2 with procaspase-8 mimics homotypic DED-DED complex formation where the conserved hydrophobic motif of procaspase-8 DED-B (F122/L123) rests in the groove formed between α2/α3 helices of E2 TAD. Strikingly, this mode of non-homotypic E2-procapase-8 binding is also similar to interactions reported for well established E2- Bromodomain-containing protein 4 (Brd4) complexes^30^, thus consistent with the role these residues play in defining this interaction surface.

The residues on E2 that map to the interface are conserved in the papillomavirus family with residue R41 absolutely conserved in all the variants, while W42 and F48 are conserved in terms of their hydrophobicity (see Supplementary Fig. S6). A recent study on HPV16-induced apoptosis demonstrated direct interaction of E2 with cFLIP, a DED-containing decoy protein which is a protease-deficient caspase-8 homolog^31^,^32^. Importantly, the proapoptotic property of E2 is rather linked to its sub-cellular localization than its inherent attributes. Unlike HR-HPVs, E2 from the low-risk HPV (LR-HPV) types lack the nuclear export signal (NES) and hence is unable to get exported from nucleus to the cytoplasm. Therefore, the LR-HPV E2 proteins remain strictly nuclear due to the presence of a dominant functional nuclear localization signal (NLS) in the hinge domain, while HR-HPV E2 shows a nucleo-cytoplasmic distribution. It was further confirmed when a cytoplasmic NLS mutant of low-risk HPV11 E2 induced apoptosis similar to that of the high-risk E2 proteins^5^. Moreover, the pro-apoptotic activity of E2 is shown to be independent of all its other virus-associated functions^11^,^12^. Altogether, this information raise an interesting possibility of the intrinsic potential of E2 proteins to bind DED-containing proteins via the identified conserved binding surface and thereby promote apoptosis, provided they localize in the cytoplasm. However, further studies with E2 proteins from low and high-risk viruses are essential to test this hypothesis.

Co-expression of E2 with procaspase-8 resulted in colocalization of the proteins in cytoplasmic punctate structures, as observed previously^33^. These structures share features similar to the proapoptotic ‘death effector filaments’ formed due to the high level expression of FADD or caspase-8 DEDs^28^. Importantly, caspase-8 activation has been proposed to depend on its DED-oligomerization induced by death receptor/FADD ligation or increase in their local concentration upon ectopic expression^29^. Oligomerization is believed to initiate proximity-induced auto-cleavage and release of active caspase-8^17^,^34^,^35^. Thus, these punctate structures may represent oligomerization of caspase-8 that has been induced upon interaction with E2, resulting into caspase activation and hence apoptosis. Recently, we reported the mechanism of classical FADD-procaspase-8 interaction and DISC formation, where FADD interacts with DED-A domain of procaspase-8 to form DED-chain crucial for caspase-8 activation^22^. Since E2 and FADD binding sites on procaspase-8 are distinct, it could be possible that FADD-mediated apoptosis occurs simultaneously or they may be mutually exclusive. Consistent with the previous report^13^, we found that E2 binds caspase-8 without any involvement of the adaptor protein FADD. Based on these evidences, we suggest that E2 influences the external cell death pathway by acting as a pseudo-adaptor protein which recruits procaspase-8 and enhances its capability to oligomerize and initiate apoptosis.

Overall, in the present study we have determined the minimal binding regions of HPV18 E2-procaspase-8 complex and the critical residues that drive this interaction. This might pave way toward designing E2 analogs with desired characteristics so as to modulate procaspase-8 activation and hence harnessing this property for disease intervention. In addition, this study contributes more directly to our understanding of this novel mechanism of caspase activation through an adaptor-independent pathway. This crucial information will not only contribute immensely to the field of apoptosis research but also toward therapeutics, thus providing an alternative strategy for apoptosis regulation.

## Methods

### Plasmids

cDNA for wild-type procaspase-8-pcDNA3.0, FADD in pET29b and pEGFPc1 HPV18 E2 full length constructs are kind gifts from Prof. G. Salvesen and Dr. F. Thierry. For co-expression and interaction studies in *E. coli*, HPV18 E2 TAD (residues 1–201) was subcloned in chloramphenicol resistant pACYC-derived plasmid vector (Clontech Laboratories, Inc.) using restriction enzymes NcoI and HindIII. A C-terminal 6X his-tag was introduced by site-directed mutagenesis, to facilitate affinity purification. Procaspase-8 DED-A (1–80), DED-B (100–181) and DED-AB (1–181) were subcloned in ampicillin resistant pMALc5E-TEV-vector (New England Biolabs) digested with BamHI and EcoRI. For expression in mammalian cells, procaspase-8 full-length and prodomain was sub-cloned in pmCherry-n1 vector. Mutations were generated using QuickChange site-directed mutagenesis kit (Stratagene) and the sequences were verified by automated DNA sequencing (ACTREC sequencing facility).

### Generation of deletion mutants

Using Quick Change kit (Stratagene), PCR-based deletion mutagenesis was performed to generate 12 deletion constructs of pMAL-c5E-TEV procaspase-8 DED-B sub-cloned between restriction sites BamHI and EcoRI. The list of primers and the residue number of the constructs are provided in the online supplemental information as Supplementary Tables S2 and S3, respectively. Sequential deletion of the helices was carried out using the respective primers on the relevant templates.

### Protein co-expression, purification and *in vitro* isolation of the complex

For co-expression, the plasmids were co-transformed in *E.coli* BL21 (DE3). The cells were grown in 2 μg/ml arabinose-supplemented LB medium (for inducing E2 TAD-his_6_) at 37 °C. Later, MBP-tagged DED construct was induced with 0.4 mM IPTG at optical density (OD_600_) = 0.6 and were grown overnight at 18 °C. For isolation of the complex, the cell lysate in buffer-A (20 mM Na_2_HPO_4_/NaH_2_PO_4_, pH 7.8, 250 mM NaCl, 0.1 mM DTT, and 1% glycerol) was passed through pre-equilibrated Ni-IDA or amylose resin and purified as described earlier^36^. The complexes were separated on 12% SDS-PAGE and immunoblotted with 1:1000 dilution of anti-E2 antibody (sc-26939, Santa Cruz Biotechnology, Inc.).

### Stability analysis of E2-DED complex

The protocol was adapted as described previously for Fas-FADD complex^23^. Briefly, isolated TAD - DED-B complex was diluted to different concentrations in the range of micromolar to nanomolar. The diluted complexes were then mixed with 50 μl of amylose resin and incubated for 1 h at 4 °C. Post wash, the bead-bound proteins were eluted with SDS sample buffer. Protein was visualized on 12% SDS-PAGE and the E2 TAD to MBP DED-B ratio was determined by densitometric analysis of background-corrected band intensity using the ImageJ software (National Institutes of Health).

### GST pull-down experiment

Equivalent amounts of bacterial lysate expressing GST, GST-E2 full length or GST-E2 TAD were incubated on Glutathione Sepharose 4B resin (GE Healthcare Life Sciences) for 1 h at room temperature. Beads were washed three times with buffer-A containing 0.5% Triton. The beads were further incubated with 50–60 μg of recombinant FADD, which was purified as described earlier^22^, for 3 h at 4 °C. Post wash, the bound proteins were separated on SDS-PAGE and were probed with 1:800 dilution of anti-his antibody (Abcam, Cambridge, USA).

### Co-immunoprecipitation assays

HEK293 cells were maintained in DMEM media supplemented with 10% foetal bovine serum (FBS). Cells were seeded in 6-well plates and co-transfected with 1.5 μg of pEGFPc1 HPV18 E2 and 500 ng of pcDNA3.0 caspase-8 vectors using Lipofectamine 2000 (Life Technologies), as per the manufacturer’s instructions. Post 24 h, the transfected cells were lysed and immunoprecipitated using 2 μg of anti-E2 or anti-FADD (sc-56093; Santa Cruz Biotechnology, Inc.) as described earlier^22^. Eluted proteins and cell lysates were separated on SDS-PAGE and immunoblotted using anti-HA (H9658; Sigma-Aldrich, USA) or anti-E2 (sc-26939, Santa Cruz Biotechnology, Inc.) antibodies.

### Confocal imaging

Cells were grown to 50–60% confluency in DMEM supplemented with 10% FBS on glass-bottomed dishes (Cell E&G, Houston, USA). Before transfection, the cells were placed in Opti-MEM (Life Technologies) and transfected with the constructs described in the Fig. 3A,B, with Lipofectamine 2000 reagent. After 20–24 h of transfection, live-cell imaging was performed at 37 °C in CO2-controlled chamber. GFP-tagged E2 and mCherry-fused procaspase-8 fluorescence were excited using a confocal system with an Argon 488-nm and helium/neon 543-nm lasers. All the images were captured using Plan-Apochromat 63 × 1.4 NA (numerical aperture) objective in 12-bit format using Zeiss LSM 510 Meta confocal laser scanning microscope. Images acquired were further processed and analysed with Image J 1.43 software (National Institutes of Health).

### Molecular modeling and docking

Structures of procaspase-8 DED-AB (1–190) and HPV18 E2 TAD were homology-modeled as described previously^22^,^36^. E2 TAD was docked on to procaspase-8 DED using HADDOCK^37^. The set of accessible interacting residues were evaluated using WHATIF^38^ and taken as the active residues, while the passive residues were defined automatically to obtain the best binding mode. The HADDOCK-generated TAD-DED complex with the best score was energy minimized and simulated as described earlier^39^. The interface residues were evaluated with PDBsum generate server^40^,^41^ and Protein Interactions Calculator^42^ using the default cut-offs.

### Circular Dichroism (CD) and Fluorescence Spectroscopy

Far-UV CD scans were acquired using a JASCO J 815 spectropolarimeter (Jasco, Easton, USA) for 10 μM proteins at 25 °C between 195 and 260 nm. The scan speed was 20 nm/min and data was averaged for 5 accumulations and mean residue ellipticity was calculated as described previously^43^. Fluorescence emission was measured for protein solutions (2 μM) using a Fluorolog-3 spectrofluorometer (Horiba Scientific, Edison, USA) with 295 nm excitation followed by emission between 310 and 400 nm.

### Caspase-8 activity assay

HEK293 cells were transfected with the plasmids as indicated in the Fig. 5B. Post 24–28 h of transfection, cells were lysed in the assay buffer and caspase activity was measured as described previously^13^. Briefly, crude extract containing 30–40 μg proteins was assayed with 200 nM of the substrate Ac-IETD-AFC (Enzo Life Sciences) in 100 μl of caspase assay buffer at 37 °C for 2 h. The reaction was monitored with a fluorescence microplate reader (Berthold Technologies) with excitation and emission wavelengths of 405 and 510 nm, respectively. The rate of IETD hydrolysis was calculated by linear regression analysis with KaleidaGraph (Synergy Software Systems). For each experiment, cells expressing wild-type GFP-E2 incubated with pan caspase inhibitor Z-VAD-FMK (40 μM) was kept as control.

### Cell viability

Post 36 h of transfection, HEK293 cells were trypsinized and washed twice with ice-cold phosphate-buffered saline (PBS). One million cells were re-suspended in PBS-10% serum containing 2 μg/μl propidium iodide (PI), and analyzed by FACS Calibr (BD Bioscience, USA) for GFP fluorescence and PI content. Percent dead cell was quantitated by GFP and PI-positive cells /GFP-positive cells.

### DNA fragmentation

A DNA fragmentation assay was performed as described previously^44^. Post 36 h of transfection, HEK293 cells (1 × 10^6^) were lysed with 100 μl lysis buffer (50 mM Tris-HCl (pH 8.0), 1% Nonidet P-40, and 20 mM EDTA), followed by the addition of 10% SDS solution and 5 μg/ml RNase A and incubation at 56 °C for 2 h. The cells were further treated with 2.5 μg/ml proteinase K for 2 h at 37 °C. After ethanol precipitation, 3 μg of DNA was electrophoresed on a 2% agarose gel, visualized with ethidium bromide and photographed under short-wave ultraviolet light.

## Additional Information

**How to cite this article**: Singh, N. *et al.* Insights into the mechanism of human papillomavirus E2-induced procaspase-8 activation and cell death. *Sci. Rep.* **6**, 21408; doi: 10.1038/srep21408 (2016).

## Supplementary Material

Supplementary Information

## References

[b1] Scheffner M., Huibregtse J. M., Vierstra R. D. & Howley P. M. The HPV-16 E6 and E6-AP complex functions as a ubiquitin-protein ligase in the ubiquitination of p53. Cell 75, 495–505 (1993). 8221889 10.1016/0092-8674(93)90384-3

[b2] Filippova M., Parkhurst L. & Duerksen-Hughes P. J. The human papillomavirus 16 E6 protein binds to Fas-associated death domain and protects cells from Fas-triggered apoptosis. J Biol Chem 279, 25729–25744 (2004). 15073179 10.1074/jbc.M401172200

[b3] Blachon S. & Demeret C. The regulatory E2 proteins of human genital papillomaviruses are pro-apoptotic. Biochimie 85, 813–819 (2003). 14585548 10.1016/j.biochi.2003.09.008

[b4] Desaintes C., Demeret C., Goyat S., Yaniv M. & Thierry F. Expression of the papillomavirus E2 protein in HeLa cells leads to apoptosis. EMBO J 16, 504–514 (1997). 9034333 10.1093/emboj/16.3.504 PMC1169654

[b5] Blachon S., Bellanger S., Demeret C. & Thierry F. Nucleo-cytoplasmic shuttling of high-risk HPV E2 proteins induces apoptosis. J. Biol. Chem. 280, 36088–36098 (2005). 16135518 10.1074/jbc.M505138200

[b6] Parish J. L. *et al.* E2 proteins from high- and low-risk human papillomavirus types differ in their ability to bind p53 and induce apoptotic cell death. J Virol 80, 4580–4590 (2006). 16611918 10.1128/JVI.80.9.4580-4590.2006 PMC1472007

[b7] Longworth M. S. & Laimins L. A. Pathogenesis of human papillomaviruses in differentiating epithelia. Microbiol Mol Biol Rev 68, 362–372 (2004). 15187189 10.1128/MMBR.68.2.362-372.2004 PMC419925

[b8] Williams V. M., Filippova M., Soto U. & Duerksen-Hughes P. J. HPV-DNA integration and carcinogenesis: putative roles for inflammation and oxidative stress. Future Virol 6, 45–57 (2011). 21318095 10.2217/fvl.10.73 PMC3037184

[b9] Adams A. K., Wise-Draper T. M. & Wells S. I. Human papillomavirus induced transformation in cervical and head and neck cancers. Cancers (Basel) 6, 1793–1820 (2014). 25226287 10.3390/cancers6031793 PMC4190568

[b10] Collins S. I. *et al.* Disruption of the E2 gene is a common and early event in the natural history of cervical human papillomavirus infection: a longitudinal cohort study. Cancer Res 69, 3828–3832 (2009). 19401452 10.1158/0008-5472.CAN-08-3099

[b11] Webster K. *et al.* The human papillomavirus (HPV) 16 E2 protein induces apoptosis in the absence of other HPV proteins and via a p53-dependent pathway. J Biol Chem 275, 87–94 (2000). 10617590 10.1074/jbc.275.1.87

[b12] Demeret C., Garcia-Carranca A. & Thierry F. Transcription-independent triggering of the extrinsic pathway of apoptosis by human papillomavirus 18 E2 protein. Oncogene 22, 168–175 (2003). 12527886 10.1038/sj.onc.1206108

[b13] Thierry F. & Demeret C. Direct activation of caspase 8 by the proapoptotic E2 protein of HPV18 independent of adaptor proteins. Cell Death Differ 15, 1356–1363 (2008). 18421300 10.1038/cdd.2008.53

[b14] Carrington P. E. *et al.* The structure of FADD and its mode of interaction with procaspase-8. Mol Cell 22, 599–610 (2006). 16762833 10.1016/j.molcel.2006.04.018

[b15] Chang D. W. *et al.* Oligomerization is a general mechanism for the activation of apoptosis initiator and inflammatory procaspases. J Biol Chem 278, 16466–16469 (2003). 12637514 10.1074/jbc.C300089200

[b16] Medema J. P. *et al.* FLICE is activated by association with the CD95 death-inducing signaling complex (DISC). EMBO J 16, 2794–2804 (1997). 9184224 10.1093/emboj/16.10.2794 PMC1169888

[b17] Shi Y. Caspase activation: revisiting the induced proximity model. Cell 117, 855–858 (2004). 15210107 10.1016/j.cell.2004.06.007

[b18] Slee E. A., Adrain C. & Martin S. J. Executioner caspase-3, -6, and -7 perform distinct, non-redundant roles during the demolition phase of apoptosis. J Biol Chem 276, 7320–7326 (2001). 11058599 10.1074/jbc.M008363200

[b19] Kumar S. & Colussi P. A. Prodomains-adaptors-oligomerization: the pursuit of caspase activation in apoptosis. Trends Biochem Sci 24, 1–4 (1999). 10087912 10.1016/s0968-0004(98)01332-2

[b20] Gervais F. G. *et al.* Recruitment and activation of caspase-8 by the Huntingtin-interacting protein Hip-1 and a novel partner Hippi. Nat Cell Biol 4, 95–105 (2002). 11788820 10.1038/ncb735

[b21] Yu J. W. & Shi Y. FLIP and the death effector domain family. Oncogene 27, 6216–6227 (2008). 18931689 10.1038/onc.2008.299

[b22] Singh N., Hassan A. & Bose K. Molecular basis of death effector domain chain assembly and its role in caspase-8 activation. FASEB J. 10.1096/fj.15-272997 (2015). 26370846

[b23] Scott F. L. *et al.* The Fas-FADD death domain complex structure unravels signalling by receptor clustering. Nature 457, 1019–1022 (2009). 19118384 10.1038/nature07606 PMC2661029

[b24] Shen C. *et al.* Crystal structure of the death effector domains of caspase-8. Biochem Biophys Res Commun 463, 297–302 (2015). 26003730 10.1016/j.bbrc.2015.05.054

[b25] Park H. H. *et al.* The death domain superfamily in intracellular signaling of apoptosis and inflammation. Annu Rev Immunol 25, 561–586 (2007). 17201679 10.1146/annurev.immunol.25.022106.141656 PMC2904440

[b26] Reichmann D., Rahat O., Cohen M., Neuvirth H. & Schreiber G. The molecular architecture of protein-protein binding sites. Curr Opin Struct Biol 17, 67–76 (2007). 17239579 10.1016/j.sbi.2007.01.004

[b27] Bogan A. A. & Thorn K. S. Anatomy of hot spots in protein interfaces. J Mol Biol 280, 1–9 (1998). 9653027 10.1006/jmbi.1998.1843

[b28] Siegel R. M. *et al.* Death-effector filaments: novel cytoplasmic structures that recruit caspases and trigger apoptosis. J Cell Biol 141, 1243–1253 (1998). 9606215 10.1083/jcb.141.5.1243 PMC2137190

[b29] Dickens L. S. *et al.* A death effector domain chain DISC model reveals a crucial role for caspase-8 chain assembly in mediating apoptotic cell death. Mol Cell 47, 291–305 (2012). 22683266 10.1016/j.molcel.2012.05.004 PMC3477315

[b30] Abbate E. A., Voitenleitner C. & Botchan M. R. Structure of the papillomavirus DNA-tethering complex E2:Brd4 and a peptide that ablates HPV chromosomal association. Mol Cell 24, 877–889 (2006). 17189190 10.1016/j.molcel.2006.11.002

[b31] Wang W. *et al.* Triggering of death receptor apoptotic signaling by human papillomavirus 16 E2 protein in cervical cancer cell lines is mediated by interaction with c-FLIP. Apoptosis 16, 55–66 (2011). 20882347 10.1007/s10495-010-0543-3

[b32] Wang W. *et al.* The relationship between c-FLIP expression and human papillomavirus E2 gene disruption in cervical carcinogenesis. Gynecol Oncol. 105, 571–577 (2007). 17433827 10.1016/j.ygyno.2007.01.051

[b33] Prikhod’ko E. A. *et al.* The NS3 protein of hepatitis C virus induces caspase-8-mediated apoptosis independent of its protease or helicase activities. Virology 329, 53–67 (2004). 15476874 10.1016/j.virol.2004.08.012

[b34] Yang X., Chang H. Y. & Baltimore D. Autoproteolytic activation of pro-caspases by oligomerization. Mol Cell 1, 319–325 (1998). 9659928 10.1016/s1097-2765(00)80032-5

[b35] Muzio M., Stockwell B. R., Stennicke H. R., Salvesen G. S. & Dixit V. M. An induced proximity model for caspase-8 activation. J Biol Chem 273, 2926–2930 (1998). 9446604 10.1074/jbc.273.5.2926

[b36] Singh N., Kanthaje S. & Bose K. Equilibrium dissociation and unfolding of human papillomavirus E2 transactivation domain. Biochem Biophys Res Commun 463, 496–503 (2015). 26091566 10.1016/j.bbrc.2015.05.057

[b37] de Vries S. J., van Dijk M. & Bonvin A. M. The HADDOCK web server for data-driven biomolecular docking. Nat Protoc 5, 883–897 (2010). 20431534 10.1038/nprot.2010.32

[b38] Vriend G. WHAT IF: a molecular modeling and drug design program. J Mol Graph 8, 52–56, 29 (1990). 2268628 10.1016/0263-7855(90)80070-v

[b39] Singh N., D’Souza A., Cholleti A., Sastry G. M. & Bose K. Dual regulatory switch confers tighter control on HtrA2 proteolytic activity. FEBS J 281, 2456–2470 (2014). 24698088 10.1111/febs.12799

[b40] Laskowski R. A., Chistyakov V. V. & Thornton J. M. PDBsum more: new summaries and analyses of the known 3D structures of proteins and nucleic acids. Nucleic Acids Res 33, D266–268 (2005). 15608193 10.1093/nar/gki001 PMC539955

[b41] de Beer T. A., Berka K., Thornton J. M. & Laskowski R. A. PDBsum additions. Nucleic Acids Res 42, D292–296 (2014). 24153109 10.1093/nar/gkt940 PMC3965036

[b42] Tina K. G., Bhadra R. & Srinivasan N. PIC: Protein Interactions Calculator. Nucleic Acids Res 35, W473–476 (2007). 17584791 10.1093/nar/gkm423 PMC1933215

[b43] Kelly S. M., Jess T. J. & Price N. C. How to study proteins by circular dichroism. Biochim Biophys Acta 1751, 119–139 (2005). 16027053 10.1016/j.bbapap.2005.06.005

[b44] Kasibhatla S. *et al.* Analysis of DNA fragmentation using agarose gel electrophoresis. CSH Protocol. 10.1101/pdb.prot4429 (2006). 22485764

