# Supplementary material for: Insights into the mechanism of human papillomavirus E2-induced procaspase-8 activation and cell death
Source: Sci Rep. 2016 Feb 24;6:21408. doi: 10.1038/srep21408 (PMC4764946; doi:10.1038/srep21408)
Supplement: Supplementary Information [file srep21408-s1.pdf]

SUPPLEMENTARY INFORMATION

Insights into the mechanism of human papillomavirus E2-induced procaspase-8 activation and cell death

Nitu Singh[1], Sanjib Senapati[2], Kakoli Bose[1]*

[1]Integrated Biophysics and Structural Biology (IBSB) Laboratory, Advanced Centre for Treatment, Research and Education in Cancer (ACTREC), Navi Mumbai, India

[2]Department of Biotechnology, Office No. 503, Lab No. 510, Indian Institute of Technology Madras, Adyar, Chennai - 600036

*Correspondence: Dr. Kakoli Bose, IBSB Lab, ACTREC, Tata Memorial Centre, Navi Mumbai 410210, India. Phone: +91 (022) 2740-5109, Fax. (022) 2740-5085, E-mail: kbose@actrec.gov.in

Figure S1

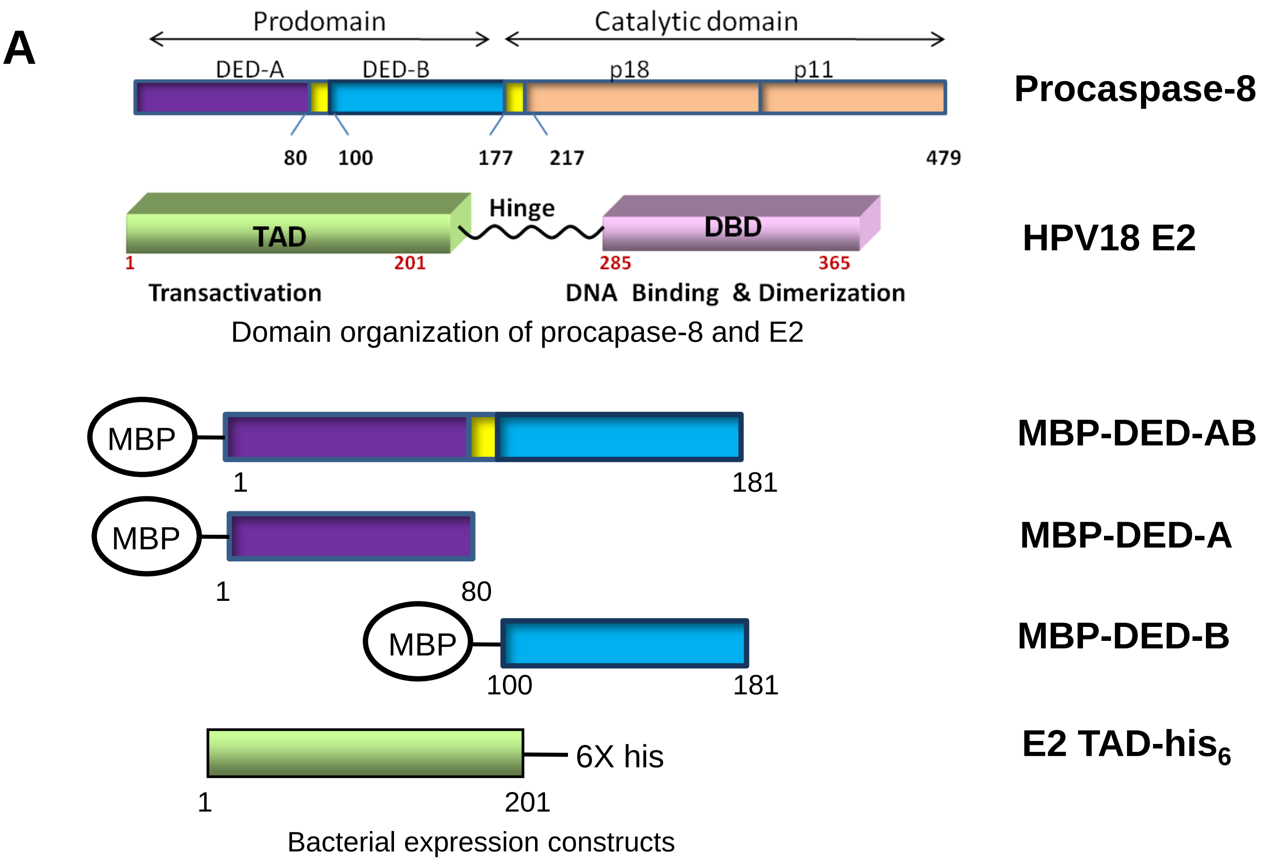
A
Prodomain
Catalytic domain
DED-A
DED-B
p18
p11
80
100
177
217
479
Procaspase-8
Hinge
TAD
DBD
1
201
285
365
HPV18 E2
Transactivation
DNA Binding & Dimerization
Domain organization of procapase-8 and E2
MBP
1
181
MBP-DED-AB
MBP
1
80
MBP-DED-A
MBP
100
181
MBP-DED-B
6X his
1
201
E2 TAD-his6
Bacterial expression constructs

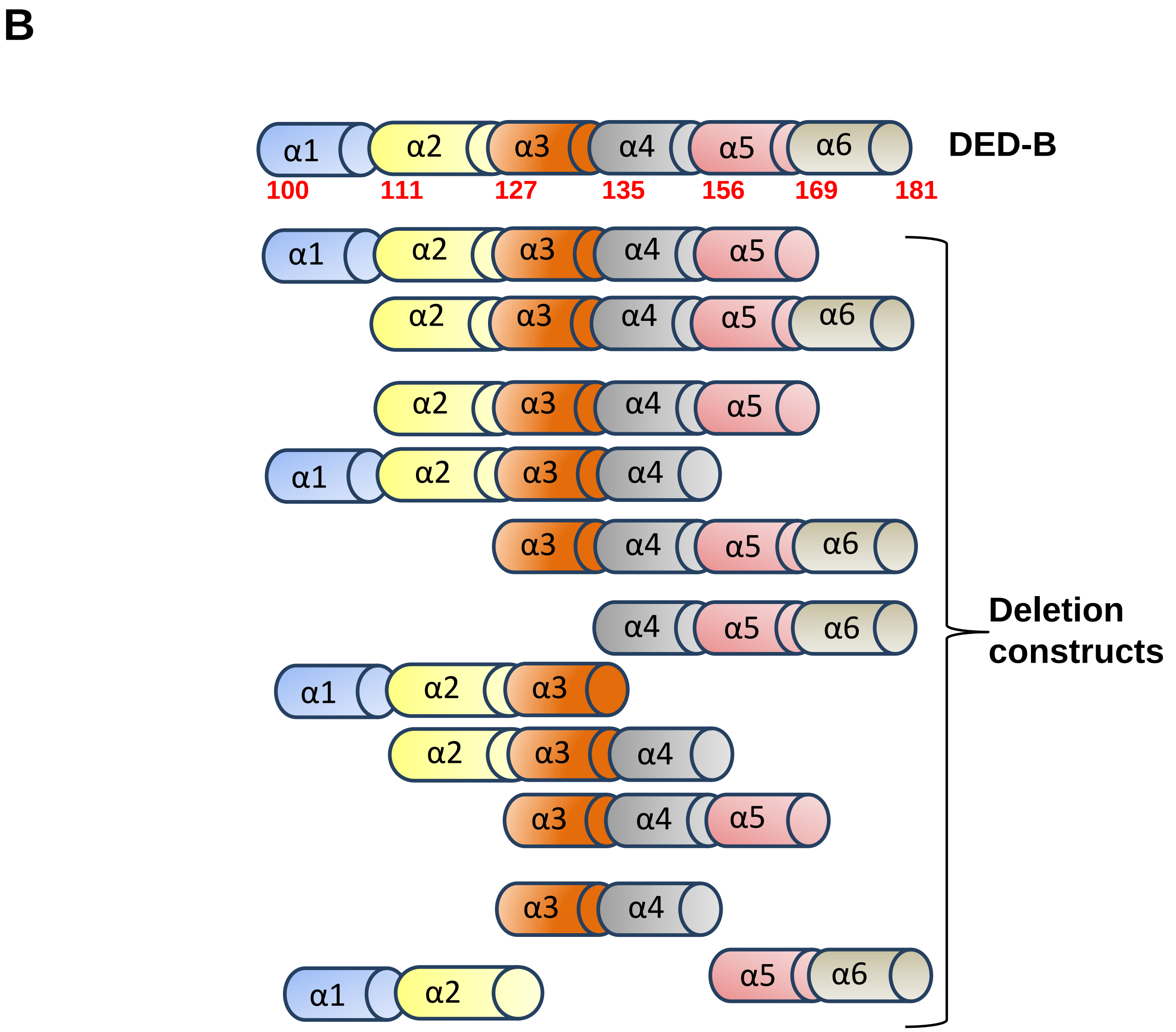
B
α1 α2 α3 α4 α5 α6
DED-B
100
111
127
135
156
169
181
Deletion constructs

Figure S2

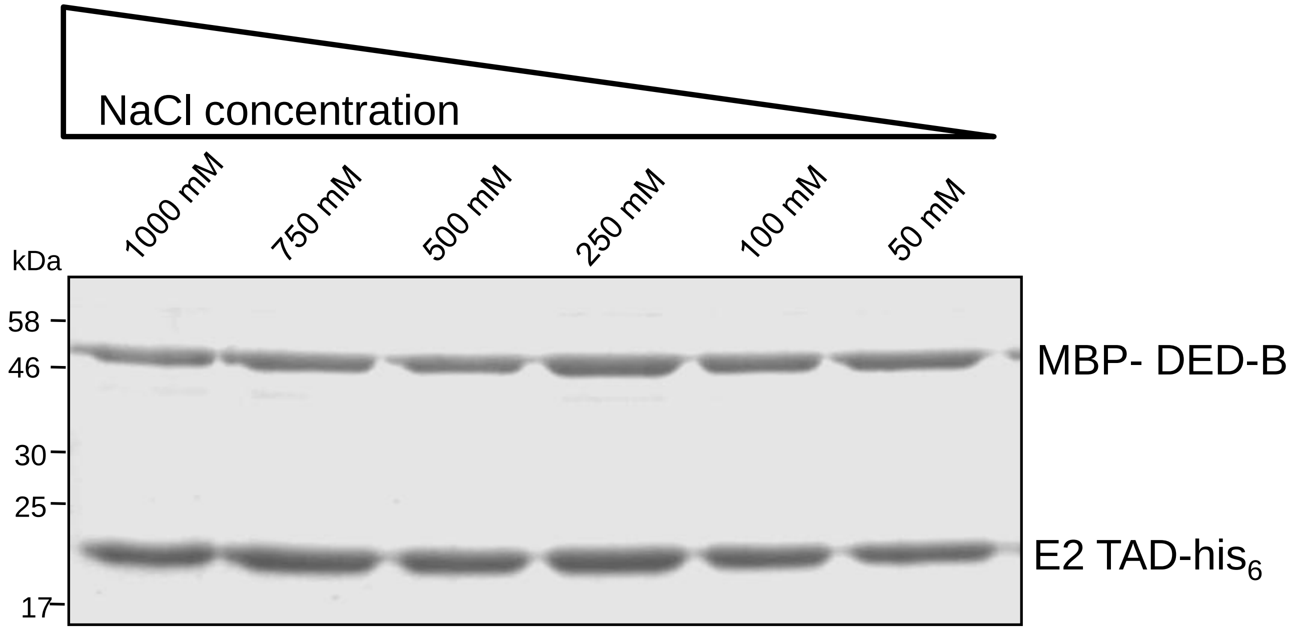

HPV18 E2 TAD – procaspase DED-B complex

Figure S3

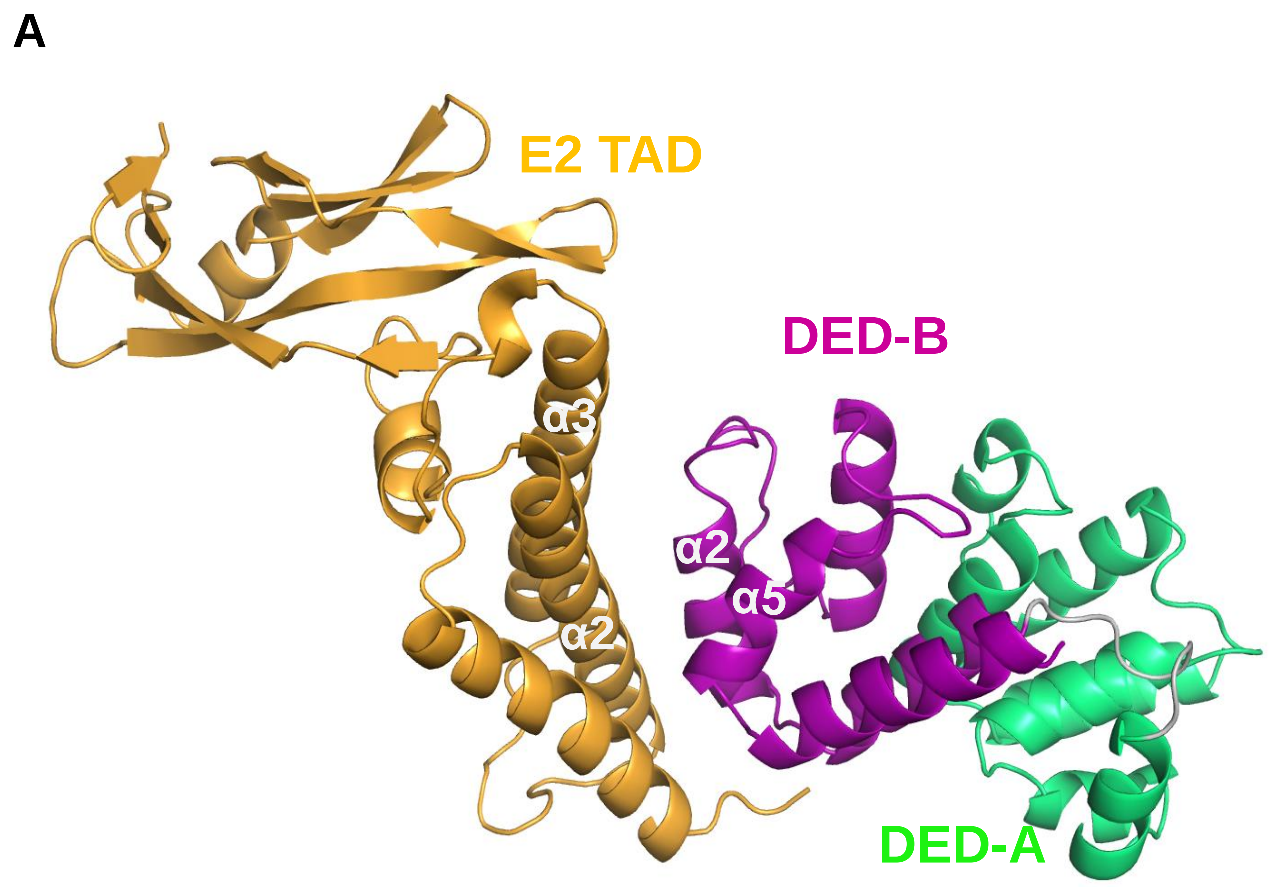
A
E2 TAD
DED-B
α3
α2
α5
α2
DED-A

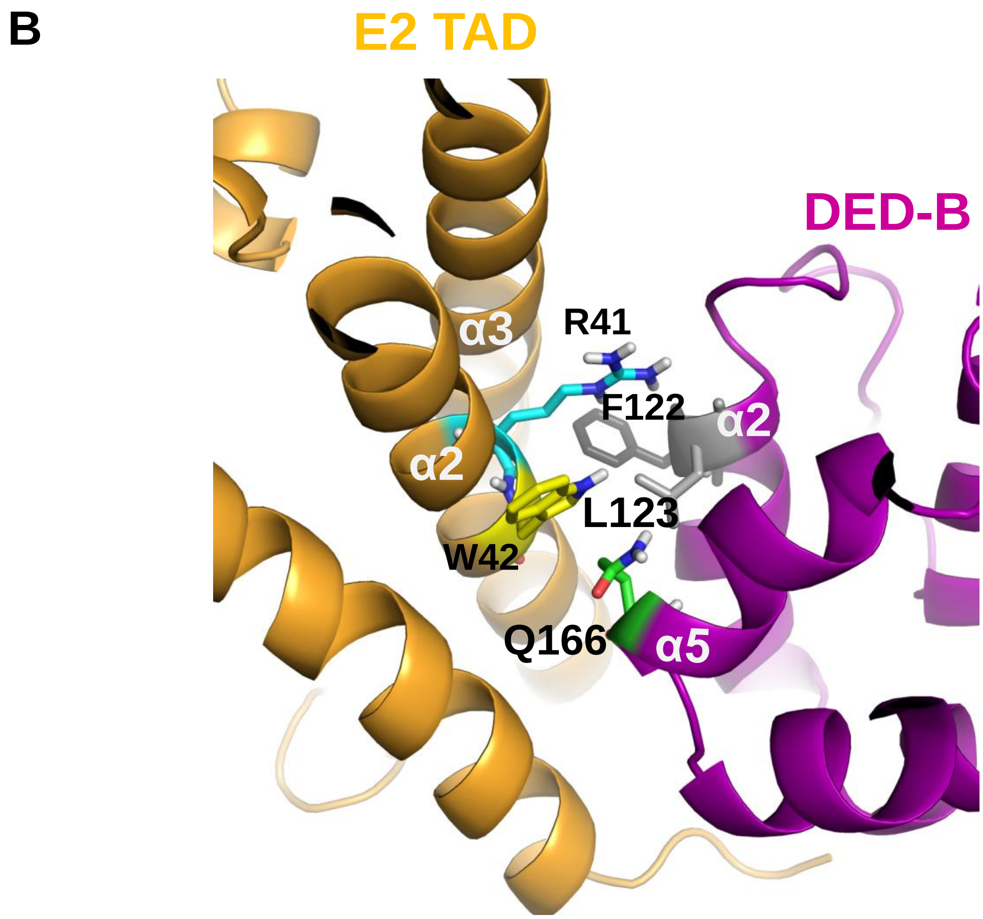
B
E2 TAD
DED-B
R41
α3
F122
α2
α2
L123
W42
Q166
α5

Figure S3

C

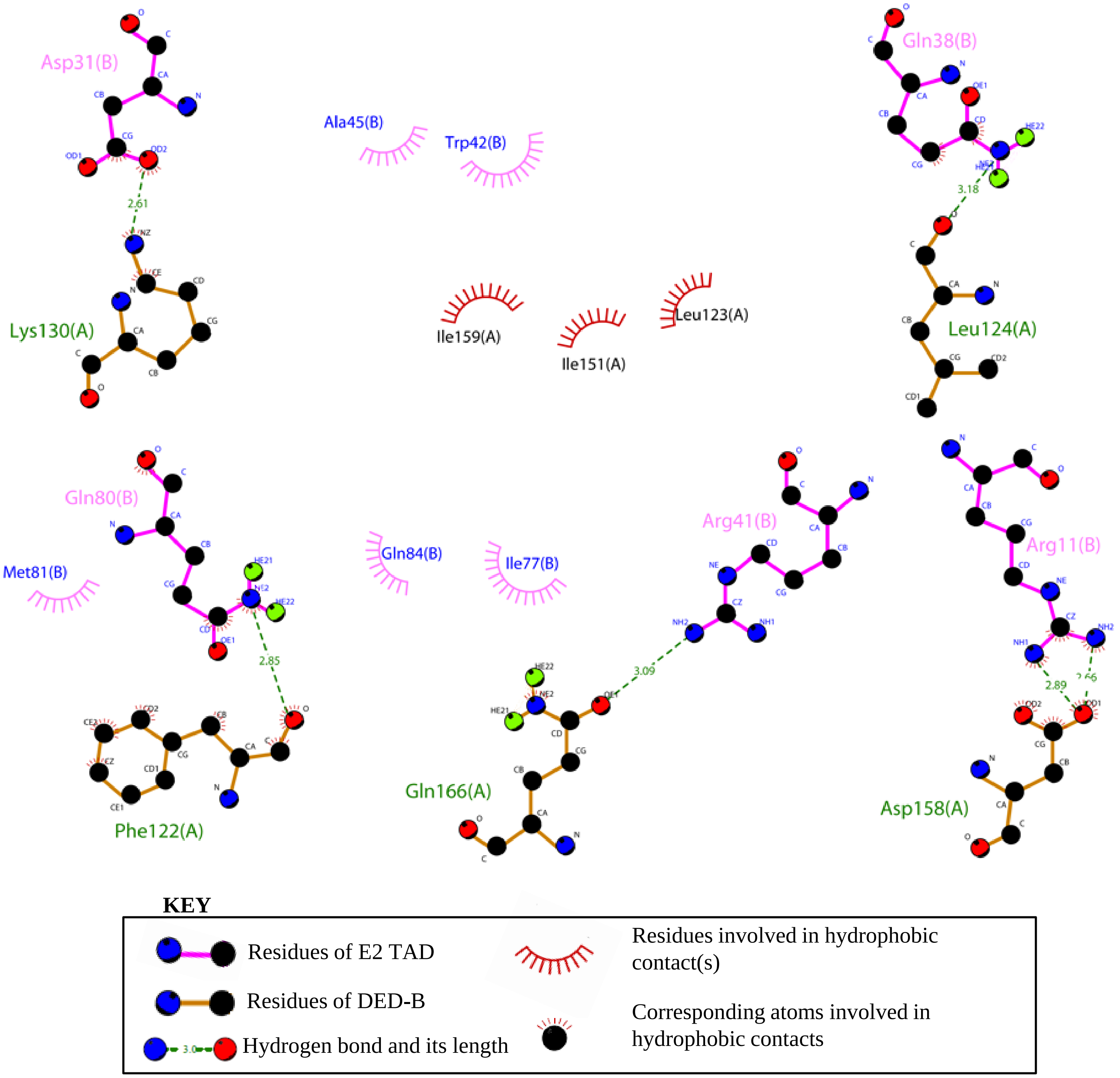

Asp31(B)
Ala45(B)
Trp42(B)
Gln38(B)
2.61
3.18
Lys130(A)
Ile159(A)
Ile151(A)
Leu123(A)
Leu124(A)
Gln80(B)
Met81(B)
Gln84(B)
Ile77(B)
Arg41(B)
Arg11(B)
2.85
3.09
2.89
Phe122(A)
Gln166(A)
Asp158(A)
KEY
Residues of E2 TAD
Residues of DED-B
3.0
Hydrogen bond and its length
Residues involved in hydrophobic contact(s)
Corresponding atoms involved in hydrophobic contacts

Figure S4

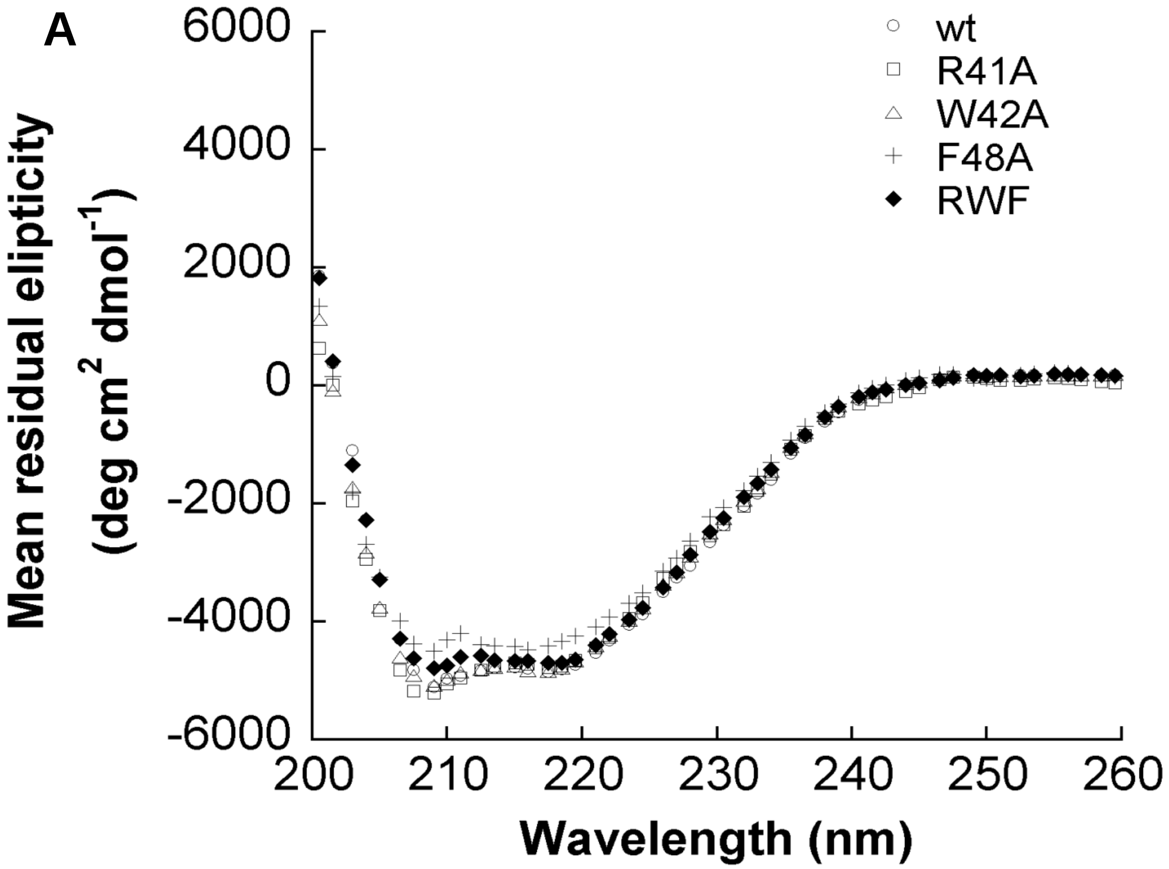
A
6000
4000
2000
0
-2000
-4000
-6000
Mean residual elipticity
(deg cm^2 dmol^{-1})
200
210
220
230
240
250
260
Wavelength (nm)
wt
R41A
W42A
F48A
RWF

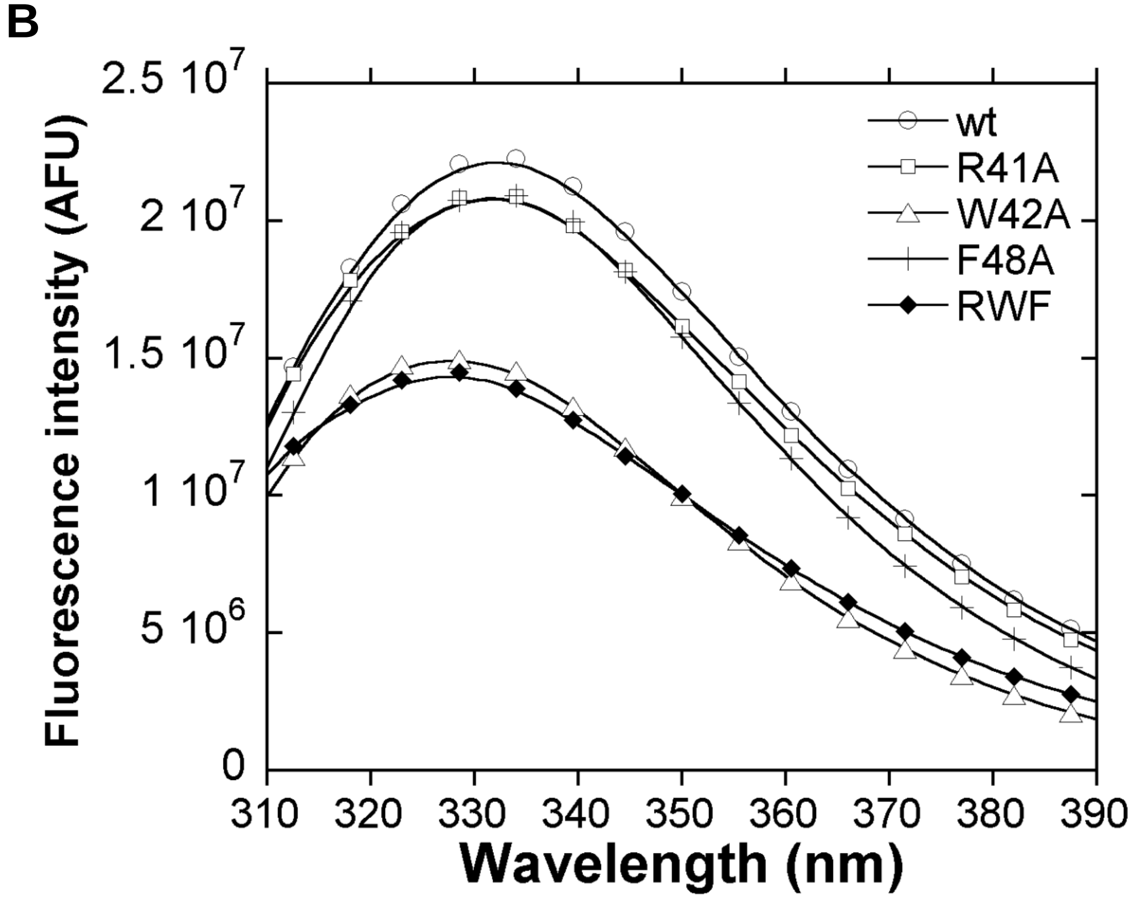
B
2.5 10^7
2 10^7
1.5 10^7
1 10^7
5 10^6
0
Fluorescence intensity (AFU)
310
320
330
340
350
360
370
380
390
Wavelength (nm)
wt
R41A
W42A
F48A
RWF

Figure S5

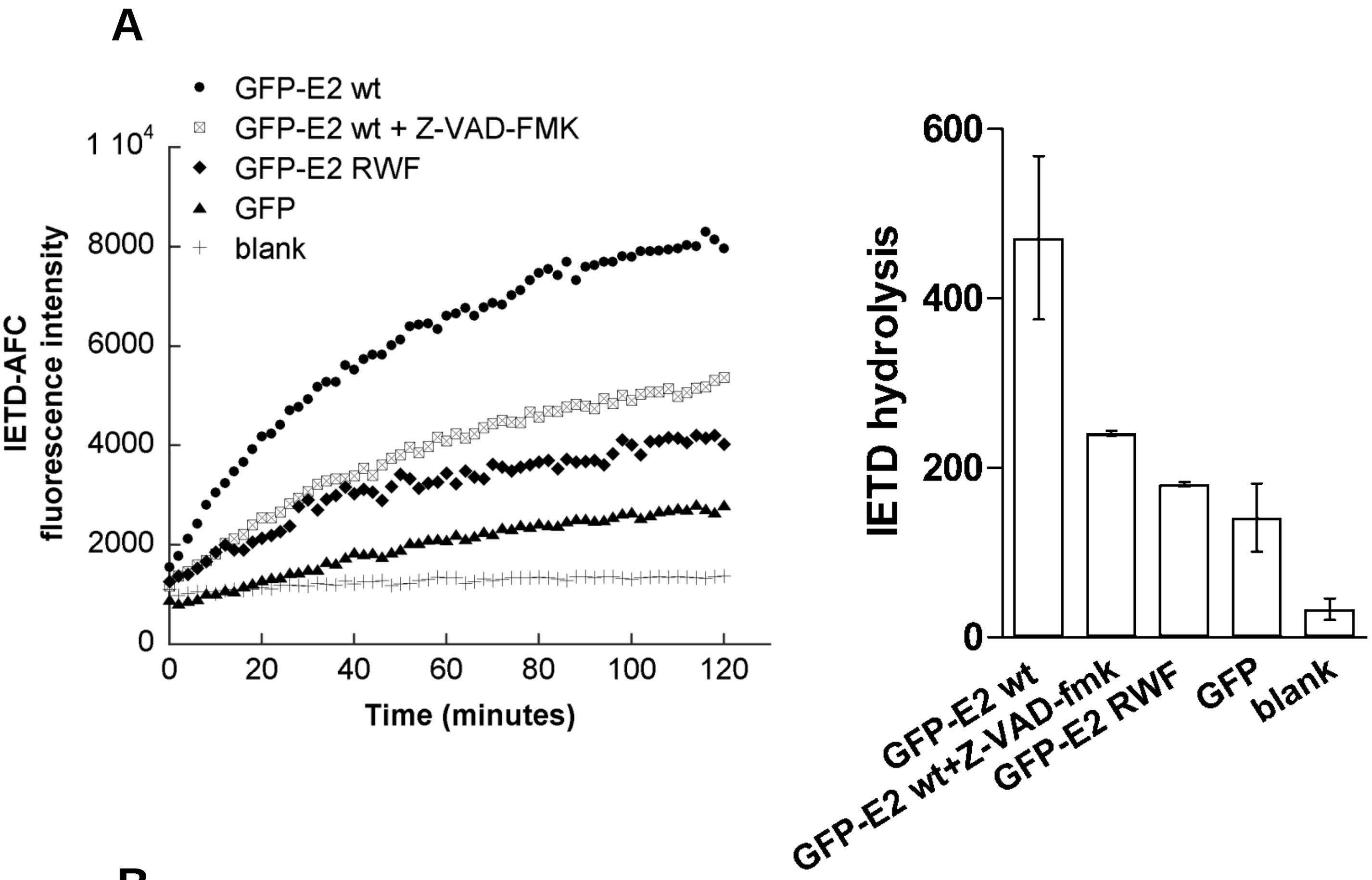
A
GFP-E2 wt
GFP-E2 wt + Z-VAD-FMK
GFP-E2 RWF
GFP
blank
IETD-AFC fluorescence intensity
1 10^4
8000
6000
4000
2000
0
0
20
40
60
80
100
120
Time (minutes)
IETD hydrolysis
600
400
200
0
GFP-E2 wt
GFP-E2 wt+Z-VAD-fmk
GFP-E2 RWF
GFP
blank

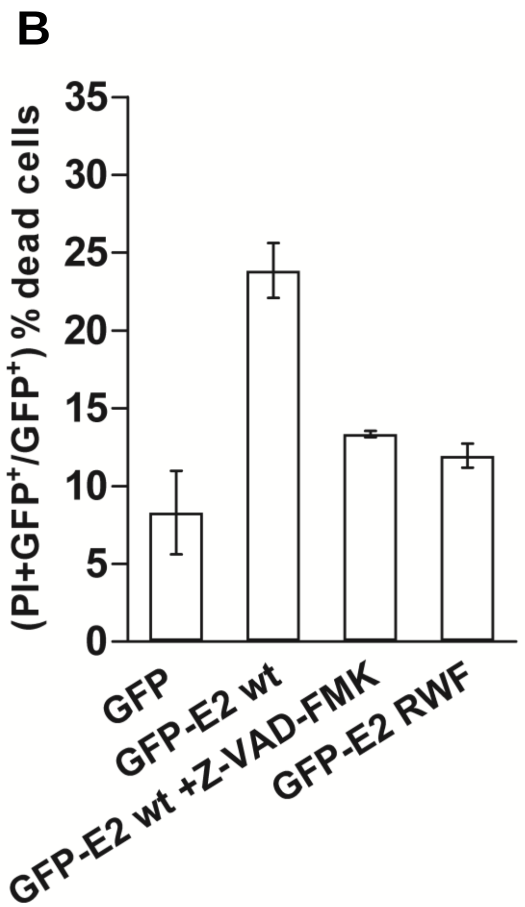
B
(PI+GFP+/GFP+) % dead cells
35
30
25
20
15
10
5
0
GFP
GFP-E2 wt
GFP-E2 wt +Z-VAD-FMK
GFP-E2 RWF

Figure S6

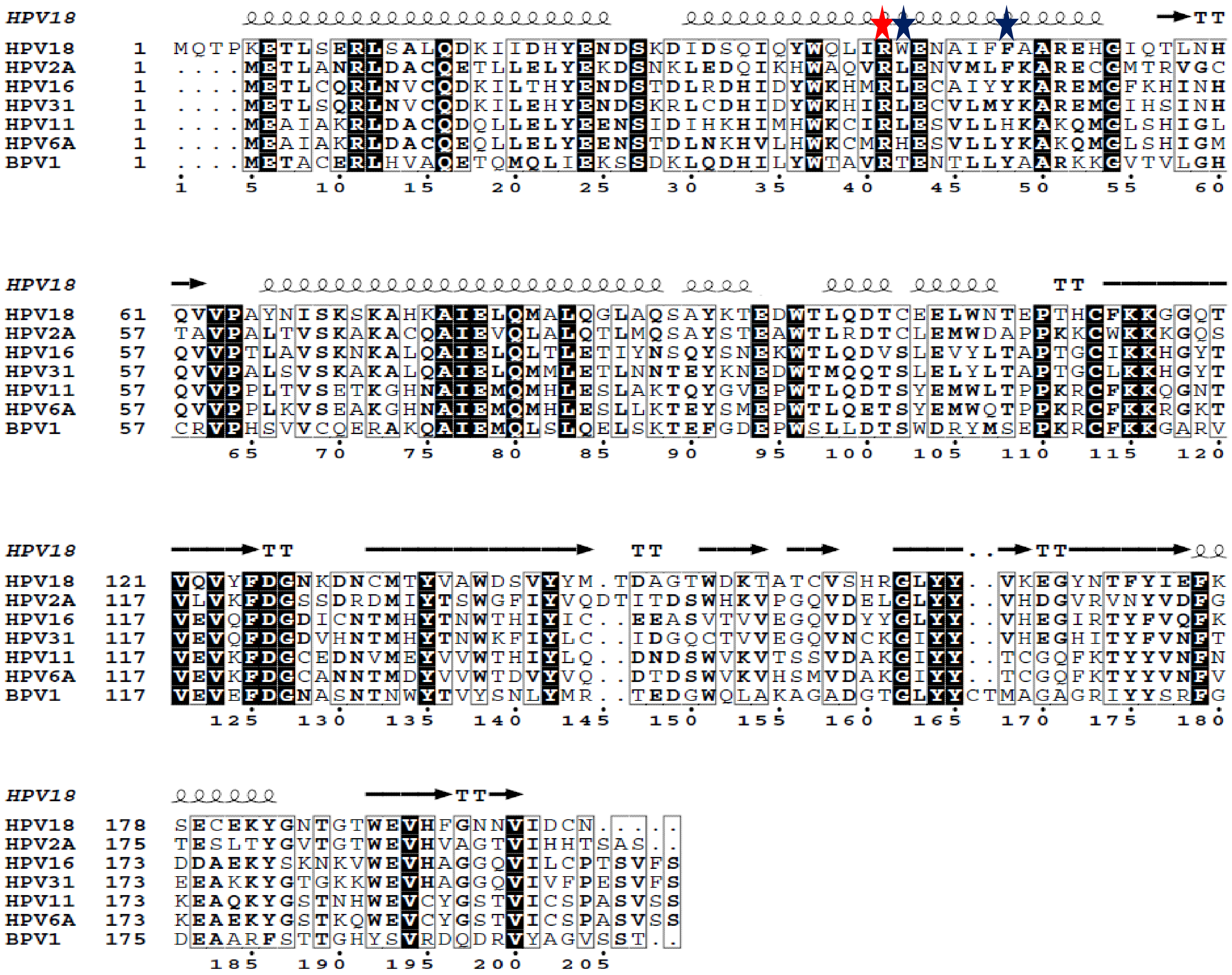
HPV18
HPV18 1 MQTPKETLSERLSALQDKIIDHYENDSKDIDSQIQYWQLIRWENAIFFAAREHGIQTLNH
HPV2A 1 ....METLANRLDACQETLLELYEKDSNKLEDQIKHWAQVRLENVMLFKARECGMTRVGC
HPV16 1 ....METLCQRLNVCQDKILTHYENDSTDLRDHIDYWKHMRLECAIYYKAREMGFKHINH
HPV31 1 ....METLSQRLNVCQDKILEHYENDSKRLCDHIDYWKHIRLECVLMYKAREMGIHSINH
HPV11 1 ....MEAIAKRLDACQDQLLELYEENSIDIHKHIMHWKCIRLESVLLHKAKQMGLSHIGL
HPV6A 1 ....MEAIAKRLDACQEQLLELYEENSTDLNKHVLHWKCMRHESVLLYKAKQMGLSHIGM
BPV1 1 ....METACERLHVAQETQMQLIEKSSDKLQDHILYWTAVRTENTLLYAARKKGVTVLGH
1 5 10 15 20 25 30 35 40 45 50 55 60
HPV18
HPV18 61 QVVPAYNISKSKAHKAIELQMALQGLAQSAYKTEDWTLQDTCEELWNTEPTHCFKKGGQT
HPV2A 57 TAVPALTVSKAKACQAIEVQLALQTLMQSAYSTEAWTLRDTCLEMWDAPPKKCWKKKGQS
HPV16 57 QVVPTLAVSKNKALQAIELQLTLETIYNSQYSNEKWTLQDVSLEVYLTAPTGCIKKHGYT
HPV31 57 QVVPALSVSKAKALQAIELQMMLETLNNTEYKNEDWTMQQTSLELYLTAPTGCLKKHGYT
HPV11 57 QVVPPLTVSETKGHNAIEMQMHLESLAKTQYGVEPWTLQDTSYEMWLTPPKRCFKKQGNT
HPV6A 57 QVVPPLKVSEAKGHNAIEMQMHLESLLKTEYSMEPWTLQETSYEMWQTPPKRCFKKRGKT
BPV1 57 CRVPHSVVCQERAKQAIEMQLSLQELSKTEFGDEPWSLLDTSWDRYMSEPKRCFKKGARV
65 70 75 80 85 90 95 100 105 110 115 120
HPV18
HPV18 121 VQVYFDGNKDNCMTYVAWDSVYYM.TDAGTWDKTATCVSHRGLYY..VKEGYNTFYIEFK
HPV2A 117 VLVKFDGSSDRDMIYTSWGFIYVQDTITDSWHKVPGQVDELGLYY..VHDGVRVNYVDFG
HPV16 117 VEVQFDGDICNTMHYTNWTHIYIC..EEASVTVVEGQVDYYGLYY..VHEGIRTYFVQFK
HPV31 117 VEVQFDGDVHNTMHYTNWKFIYLC..IDGQCTVVEGQVNCKGIYY..VHEGHITYFVNFT
HPV11 117 VEVKFDGCEDNVMEYVVWTHIYLQ..DNDSWVKVTSSVDAKGIYY..TCGQFKTYYVNFN
HPV6A 117 VEVKFDGCANNTMDYVVWTDVYVQ..DTDSWVKVHSMVDAKGIYY..TCGQFKTYYVNFV
BPV1 117 VEVEFDGNASNTNWYTVYSNLYMR..TEDGWQLAKAGADGTGLYYCTMAGAGRIYYSRFG
125 130 135 140 145 150 155 160 165 170 175 180
HPV18
HPV18 178 SECEKYGNTGTWEVHFGNNVIDCN.....
HPV2A 175 TESLTYGVTGTWEVHVAGTVIHHTSAS..
HPV16 173 DDAEKYSKNKVWEVHAGGQVILCPTSVFS
HPV31 173 EEAKKYGTGKKWEVHAGGQVIVFPESVFS
HPV11 173 KEAQKYGSTNHWEVCYGSTVICSPASVSS
HPV6A 173 KEAEKYGSTKQWEVCYGSTVICSPASVSS
BPV1 175 DEAARFSTTGHYSVRDQDRVYAGVSST..
185 190 195 200 205

SUPPLEMENTARY FIGURE LEGEND

Figure S1:

Schematic representation of the constructs. A) *Top panel* - Domain organization of procaspase-8 and HPV18 E2 proteins. *Lower panel* – Diagrammatic representation of the bacterial expression constructs for procaspase-8 prodomain (containing DED-A and DED-B) and transactivation domain of E2. B) Design of 12 deletion mutants of DED-B. The individual helices are shown in different colors. DED: Death effector domain, TAD: Transactivation domain, DBD: DNA binding domain, MBP: Maltose binding protein

Figure S2:

Analysis of the stability of E2-DED complex in buffers of different ionic strengths. Using amylose resin, E2 TAD – DED-B complex was isolated in buffers of different NaCl concentration ranging from 50 mM to 1000 mM. 40 μg of the complex was resolved on 12% SDS PAGE.

Figure S3:

Docking of E2-TAD and procaspase-8 tandem DED. A) E2 TAD was docked on to procaspase-8 DED using HADDOCK [1]. The resultant model revealed that α2/ α5 helices of procaspase-8 DED-B form contacts with α2/α3 helices of E2 TAD. The figure has been generated using the PyMol (http://www.pymol.org). B) Binding interface of the E2-DED complex and the interacting residues are shown as sticks. C) Dimplot of the docked E2 – procaspase-8 complex. Residues from E2 TAD and procaspase-8 DED-B are shown in pink and orange colors respectively.

Figure S4:

Secondary and tertiary structural analyses of E2 TAD wild-type and mutant proteins. A) Far ultraviolet circular dichroism spectra of E2 wild-type and mutant proteins. The measurements were made at 25 ^{o}C for 10 μM of protein solution in buffer 10 mM NaH_2PO_4/Na_2HPO_4 pH 8.0, 100 mM NaCl. Contributions to the spectra by the buffer were subtracted using control scans. All the curves are represented as mean residual ellipticity in unit deg cm^2 mol^{-1}. The CD spectra reveal that the mutants have similar secondary structure characteristics as the wild-type protein. B) Fluorescence emission scans recorded for 2 μM of wild-type and mutant E2 TAD using an excitation of 295 nm and emission in the range of

310-400 nm. Note that the emission maximum blue shifted by 3 nm (with respect to wild-type) in case of the single W42A and triple R41A, W42A, F48A (RWF) mutants most likely due to the substitution of the surface exposed W42.

Figure S5:

E2-induced cell death analyses in HeLa cell. A) Caspase-8 activity assay. Crude cell lysate of HeLa cells transfected with the indicated plasmids was analyzed for rate of hydrolysis of caspase-8 specific fluorogenic substrate IETD-AFC. *Left panel* – The experimental data obtained as the measure of caspase-8 proteolytic activity by monitoring increase in the fluorescence intensity (due to release of AFC) over the reaction time of 2 h. *Right panel* – The plot for rate of IETD hydrolysis calculated by linear regression analysis of the experimental data. The bars and the error bars represent mean and standard deviation respectively. B) Cell viability assay. Percentage of GFP-positive dead cells expressing the indicated plasmids was quantified by propidium iodide uptake.

Figure S6:

Multiple sequence alignment of E2 proteins using ESPript [2]. The asterisk highlights the residue critical for E2 – procaspase-8 interaction. BPV – Bovine papillomavirus, HPV – Human papillomavirus. HPV16, 18, 31 are high-risk while HPV2a, 6a, 11 belong to low-risk types.

Table S1 List of binding interface residue areas [Å^2] (hydrophilic, hydrophobic, and total) calculated with the POPS server [3].

**Residue**	**Hydrophobicity (Å^2)**	**Hydrophilicity (Å^2)**	**Total (Å^2)**	**% SASA[a]**
Gln35	25.25	70.98	96.22	0.46
Gln38	8.47	41.66	50.13	0.24
Arg41	9.92	61.59	71.51	0.26
Trp42	76.07	11.22	87.29	0.45
Phe48	43.79	1.80	45.59	0.27
Glu52	20.55	63.82	84.37	0.42
His53	70.45	57.73	128.1	0.70

[a]Solvent Accessibility Surface Area

Table S2 List of forward primers for deletion mutagenesis of procaspase-8 DED-B to generate 12 deletion mutants comprising different combination of α-helices. For all the mutations, the reverse primer is the complementary sequence.

**Helix-1**	GGGAGAATCTTTATTTTCAGGGC**GGATCC**ATGGAAGTGAGCAGATCAGAATTGAG
**Helix-2**	GAGAATCTTTATTTTCAGGGC**GGATCC**ATGGAAATCTCCAAATGCAAACTGGATG
**Helix-3**	GAGAATCTTTATTTTCAGGGC**GGATCC**ATGGATGACATGAACCTGCTGGATATTTTCA
**Helix-4**	CAAATGCAAACTGGATGATGACATGTGA*GAATTC*GAAGCTTGGCACTGGC
**Helix-5**	GTCATCCTGGGAGAAGGAAAGTTGTGA*GAATTC*GAAGCTTGGCACTGG
**Helix-6**	GAGTCTGTGCCCAAATCAACAAGTGA*GAATTC*GAAGCTTGGCACTGG

The text highlighted in bold and italics marks the restriction sites BamHI and EcoRI respectively. The underlined text represent the nucleotides from pMAL-c5-E-TEV vector backbone.

Table S3 The residue numbers of the procaspase-8 DED-B deletion mutants.

DED-B helices	Residue number
**α1-α2- α3- α4- α5- α6** (wt)	100 - 181
**α2- α3- α4- α5- α6**	111 - 181
**α1-α2- α3- α4- α5**	100 - 169
**α2- α3- α4- α5**	111-169
**α1-α2- α3- α4**	100 -156
**α3- α4- α5- α6**	127 - 181
**α4- α5- α6**	135 - 181
**α1-α2- α3**	100 - 137
**α2- α3- α4**	111-156
**α3- α4- α5**	127 - 169
**α3- α4**	127 - 156
**α5- α6**	156 - 181
**α1-α2**	100 - 129

REFERENCES

1. de Vries, S.J., van Dijk, M. & Bonvin, A.M. The HADDOCK web server for data-driven biomolecular docking. *Nat Protoc* **5**, 883-97 (2010).
2. Robert, X. & Gouet, P. Deciphering key features in protein structures with the new ENDscript server. *Nucleic Acids Res* **42**, W320-4 (2014).
3. Cavallo, L., Kleinjung, J. & Fraternali, F. POPS: A fast algorithm for solvent accessible surface areas at atomic and residue level. *Nucleic Acids Res* **31**, 3364-6 (2003).
